# siRNA-Mediated Gene Targeting in *Aedes aegypti* Embryos Reveals That *Frazzled* Regulates Vector Mosquito CNS Development

**DOI:** 10.1371/journal.pone.0016730

**Published:** 2011-01-31

**Authors:** Anthony Clemons, Morgan Haugen, Christy Le, Akio Mori, Michael Tomchaney, David W. Severson, Molly Duman-Scheel

**Affiliations:** 1 Department of Biological Sciences and Eck Institute for Global Health, University of Notre Dame, Notre Dame, Indiana, United States of America; 2 Department of Medical and Molecular Genetics, Indiana University School of Medicine, South Bend, Indiana, United States of America; Universidade Federal do Rio de Janeiro, Brazil

## Abstract

Although mosquito genome projects uncovered orthologues of many known developmental regulatory genes, extremely little is known about the development of vector mosquitoes. Here, we investigate the role of the Netrin receptor *frazzled (fra)* during embryonic nerve cord development of two vector mosquito species. Fra expression is detected in neurons just prior to and during axonogenesis in the embryonic ventral nerve cord of *Aedes aegypti* (dengue vector) and *Anopheles gambiae* (malaria vector). Analysis of *fra* function was investigated through siRNA-mediated knockdown in *Ae. aegypti* embryos. Confirmation of *fra* knockdown, which was maintained throughout embryogenesis, indicated that microinjection of siRNA is an effective method for studying gene function in *Ae. aegypti* embryos. Loss of *fra* during *Ae. aegypti* development results in thin and missing commissural axons. These defects are qualitatively similar to those observed in *Dr. melanogaster fra* null mutants. However, the *Aa. aegypti* knockdown phenotype is stronger and bears resemblance to the *Drosophila commissureless* mutant phenotype. The results of this investigation, the first targeted knockdown of a gene during vector mosquito embryogenesis, suggest that although Fra plays a critical role during development of the *Ae. aegypti* ventral nerve cord, mechanisms regulating embryonic commissural axon guidance have evolved in distantly related insects.

## Introduction

Completion of the *Aedes aegypti* and *Anopheles gambiae* genome projects uncovered orthologues of many known developmental regulatory genes in these two important mosquito vectors of dengue and malaria, respectively [1], [2]. Although characterization of the function of these genes could provide insight into the evolution of insect development or potentially reveal novel strategies for vector control, extremely little is known about the genetic regulation of mosquito development [3], [4]. Excellent descriptive analyses of *Ae. aegypti* embryogenesis were completed in the 1970's [5], [6], and additional developmental analyses in this species were recently published [7], [8]. Still, expression of only a handful of mosquito embryonic genes has been described in *Ae. aegypti* or other vector mosquitoes [9], [10], [11], [12], [13], [14], [15], [16]. This is likely a result of the technical challenges historically encountered by those performing developmental analyses in mosquitoes. In fact, Christophers [17], author of the most comprehensive text on the biology of *Ae. aegypti,* indicated that the eggs of this species are not the most suitable form on which to study mosquito embryology.

Given the many known advantages of studying the biology of *Ae. aegypti* [3], [18], we recently published a series of protocols for the study of its development [19], [20], [21], [22], [23]. These methodologies, in addition to those published previously [9], [11], will promote analysis of mosquito developmental genetics. We are presently employing these techniques to examine mosquito nervous system development. Analysis of mosquito neural development will lead to a better understanding of the developmental basis of motor function, sensory processing, and behavior, key aspects of mosquito host location.

During *Drosophila melanogaster* nervous system development, midline cells secrete guidance molecules such as Netrin (Net) proteins that regulate the growth of commissural axons [24], [25], [26]. The *Dr. melanogaster* Net proteins are expressed at the midline and are required for proper commissural axon guidance in the embryonic ventral nerve cord. Frazzled (Fra), the *Drosophila* homolog of the vertebrate Deleted in Colorectal Cancer (DCC) Net receptor, guides axons in response to Net signaling [27] and also controls Net distribution in flies [28]. Previous studies indicated that deletion of *netA* and *B* or *fra* results in defective guidance of commissural axons in *Drosophila* [27], [29], [30]. More recent data suggest that *Drosophila* Nets function as short-range guidance cues that promote midline crossing [31].

Although data support the homology of axon-guiding midline cells [16], [32], [33], [34], [35], [36], homology of midline cells, which form differently in various arthropod species (discussed in [32]) has been debated. To address whether common molecular mechanisms regulate nerve cord formation during arthropod nervous system development, we recently analyzed patterns of axon tract formation and the putative homology of midline cells in distantly related arthropods. These comparative analyses were aided by a cross-reactive antibody generated against the Netrin (Net) protein, a midline cell marker and regulator of axonogenesis [16]. Despite divergent mechanisms of midline cell formation and nerve cord development in arthropods, detection of conserved Net accumulation patterns suggests that Net-Fra signaling plays a conserved role in the regulation of ventral nerve cord development of Tetraconata [16]. Here, we continue to examine this hypothesis through examination of the expression of the Net receptor *frazzled* in both *Ae. aegypti* and *An. gambiae.* Moreover, for the first time, we use siRNA-mediated knockdown to functionally test this hypothesis in *Ae. aegypti.*

## Results and Discussion

### Development of the mosquito embryonic ventral nerve cord

**Figure 1:**
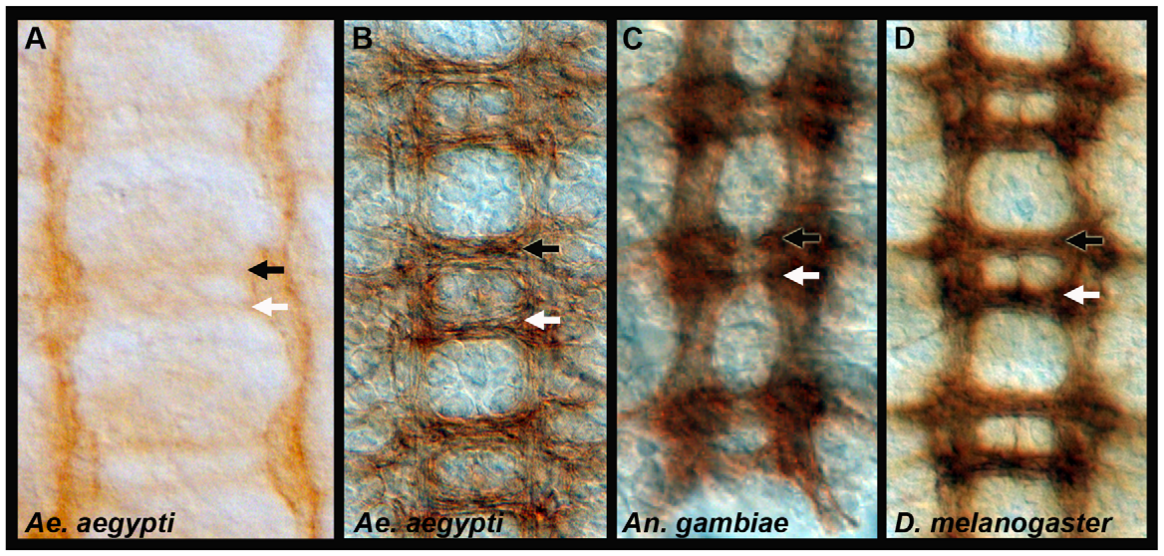
Development of the *Ae. aegypti* embryonic ventral nerve cord. Anti-acetylated tubulin staining (A–C) marks the developing axon tracts in 52 hr. (A) and 56 hr. (B) *Ae. aegypti* embryos. By 56 hrs. (C), the *Ae. aegypti* nerve cord resembles that of a 33 hr. *An. gambiae* embryo and a St. 16 *Dr. melanogaster* nerve cord (BP102 staining is shown in D). These time points in the three respective species correspond to germ-band retracted embryos in which segmentation is obvious and organogenesis has initiated. Filleted nerve cords are oriented anterior up in all panels. The anterior commissure is marked by a black arrowhead, and a white arrowhead marks the posterior commissure.

A scaffold of axon pathways develop in *Dr. melanogaster* and give rise to the embryonic ventral nerve cord, which has a ladder-like appearance (Fig. 1D). Within each segment of the developing fruit fly embryo, a pair of bilaterally symmetrical longitudinal axon tracts are pioneered separately on either side of the midline in each segment. A number of early growth cones project only on their own side, but most CNS interneurons will project their axons across the midline in either the anterior or posterior commissural axon tracts before extending rostrally or caudally in the developing longitudinals ([24], [25]; Fig 1D). Nerve cord development was assessed during mosquito embryogenesis with an anti-acetylated tubulin antibody (Fig. 1A–C). Acetylated tubulin is first detected in *Ae. aegypti* at 52 hrs. after egg laying (AEL) when the longitudinal axon tracts have begun to form and the commissural axon tracts are initiating (Fig. 1A). During the next several hours, the axon tracts thicken as additional neurons project their axons (Fig. 1B). Anterior and posterior commissures are initially fused (not shown), as observed in *Dr. melanogaster* [37]. At 56 hrs. AEL, the commissures have separated, and the mature ventral embryonic nerve cord of *Ae. aegypti* (Fig. 1B) resembles that of *An. gambiae* (33 hrs. AEL shown in Fig. 1C) and a St. 16 *Drosophila* embryo (Fig. 1D).

### Expression of *fra* in the developing mosquito CNS

**Figure 2:**
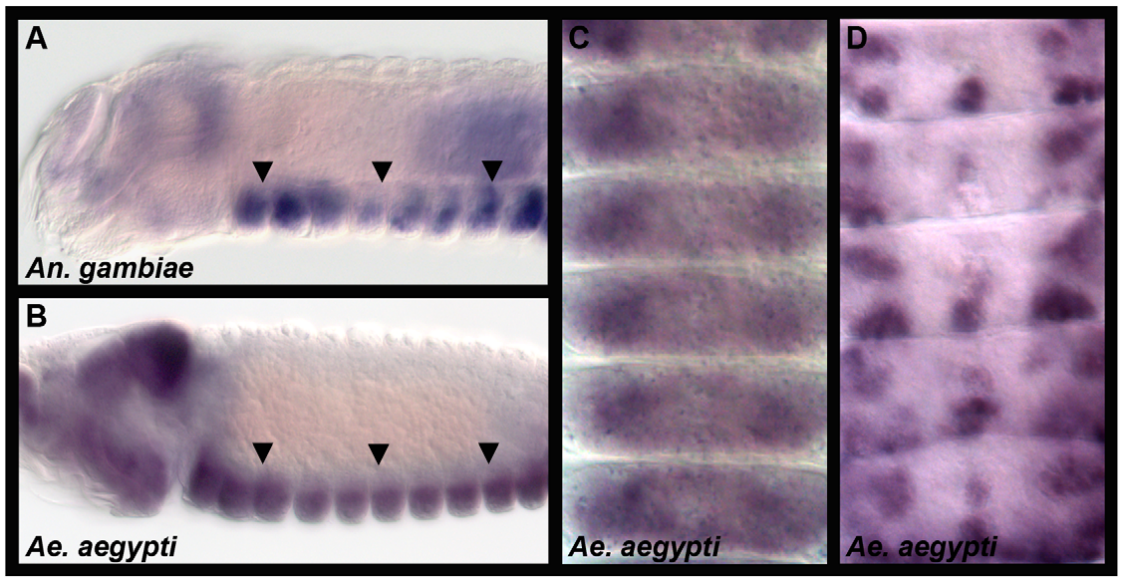
Expression of *fra* in the developing mosquito CNS. Comparable *fra* expression patterns are detected in lateral views of the developing nervous systems (arrows) of *An. gambiae* (33 hrs., A) and *Ae. aegypti* (52 hrs., B). Ventral views of *Aae fra* expression in 52 hr. (C, segments T3–A5) and 54 hr. (D; segments A2–A6) *Ae. aegypti* embryos are shown. Anterior is oriented left in A and B and up in C and D.

Net accumulation data have indicated that Net-Fra signaling may play conserved roles during insect ventral nerve cord development [16], [36]. However, in insects, *fra* expression has not been examined outside of *Drosophila*, where it is expressed on developing axons of the commissural and longitudinal axon pathways, including the earliest commissural axons [27]. Expression of *Ae. aegypti fra (Aae fra)* and *An. gambiae fra (Aga fra)* were therefore analyzed through whole-mount *in situ* hybridization at the onset of nerve cord development in both species. *Aae fra* expression initiates in developing neurons, including the earliest commissural axons, just prior to establishment of the axonal scaffold and is maintained during ventral nerve cord formation (Fig. 2B–D). Comparable *fra* expression patterns are detected in the developing nervous system of *An. gambiae* (Fig. 2A). These data are consistent with the hypothesis that Fra functions to regulate growth of commissural axons in mosquitoes.

### si-RNA mediated knockdown of *fra* during *Ae. aegypti* development

Analysis of *fra* expression (Fig. 2) suggested that this gene may regulate ventral nerve cord development in mosquitoes. Functional testing of this hypothesis required the development of a strategy to selectively inhibit gene function during mosquito development. RNA interference (RNAi) technology, which has emerged as an effective method for inhibiting gene function in many organisms, was therefore combined with previously described *Ae. aegypti* microinjection techniques [38], [39] to knockdown *fra* during *Ae. aegypti* development. Two separate siRNAs corresponding to different regions of *Aae fra, fra* siRNA-A and *fra* siRNA-B, as well as a scrambled control version of siRNA-A, were used in these experiments.

**Figure 3:**
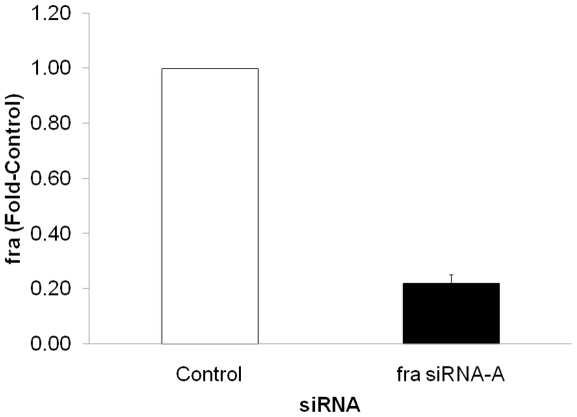
Confirmation of *fra* knockdown in *Ae. aegypti.* qRT-PCR was used to assess *fra* levels following microinjection of *fra* siRNA-A. A scrambled version of *fra* siRNA-A was injected as a control. At 72 hrs. post injection, levels of *fra* were 80% less than that of the control-injected group (N = 3, p<0.0001).

**Figure 4:**
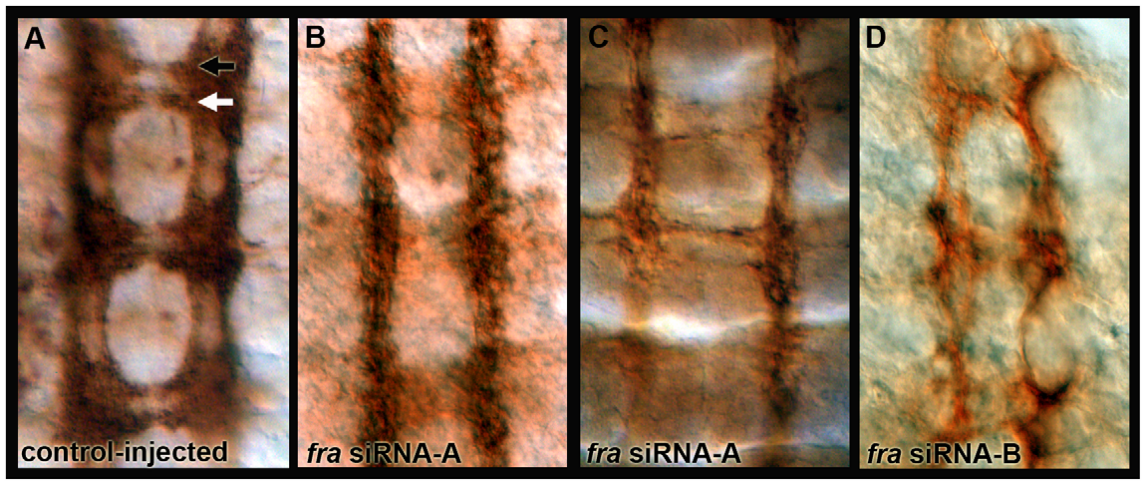
*Ae. aegypti fra* knockdown CNS phenotypes. Anti-acetylated tubulin staining (reddish brown) marks the axons of the ventral nerve cords of scrambled control (A) and *fra* siRNA injected embryos (B–D). Knockdown phenotypes characterized by thinning or loss of commissural axons were observed at 54 hrs. (B–D). Comparable results were obtained with two different siRNAs (*fra* siRNA-A in B,C; *fra* siRNA-B in D). Knockdown of *fra* was confirmed by double-labeling to detect *fra* mRNA expression (dark blue in C). Nerve cords are oriented anterior up in each panel. The anterior commissure is marked by a black arrowhead, and a white arrowhead marks the posterior commissure.

siRNAs were injected pre-cellular blastoderm, and knockdown was assessed through both quantitative real-time PCR (qRT-PCR) and whole-mount *in situ* hybridization. Multiple qRT-PCR replicates at three different time points, including 24, 48 (not shown), and 72 hrs. (Fig. 3), confirmed knockdown of *fra* that was maintained through the end of embryogenesis. At 72 hrs., the time point that was typically assayed once injection protocols and knockdown strategies had been optimized, *fra* transcript levels were reduced by 80% on average (Fig. 3, p<0.0001), and a maximum of 90% knockdown was achieved in one replicate. Knockdown in the developing CNS was verified through *in situ* hybridization, which confirmed reduced levels of *fra* transcripts in the embryonic CNS at levels comparable to those detected by qRT-PCR, and which revealed nearly complete knockdown in the developing nervous systems of embryos bearing strong phenotypes (Fig. 4C). These studies suggest that siRNA methodology can be used for targeted disruption of embryonic gene function in *Ae aegypti.*

### *Ae. aegypti fra* knockdown CNS phenotypes

The impact of *fra* knockdown on *Ae. aegypti* embryonic nerve cord development was assessed through anti-acetylated tubulin staining at 54 hrs. AEL. In embryos injected with *fra* siRNA-A, 71% of anterior commissures and 80% of posterior commissures are thin or absent (Fig. 4B, C, Table 1). As observed in *Drosophila* [27], the posterior commissure is more severely disrupted than the anterior, with 51% of the embryos displaying a severe phenotype in the posterior commissure and 36% of embryos displaying a severe anterior commissure phenotype (Table 1). Occasional breaks in the longitudinal tracts were also noted in *fra* knockdown embryos. Injection of either *fra* siRNA-A (Fig. 4B,C) or siRNA-B (Fig. 4D), which correspond to two separate *Aae fra* sequences, produced similar phenotypes. This result indicates that the knockdown phenotypes described are due to loss of *fra* and are not the result of off-site targeting. Injection of the scrambled control siRNA did not disrupt nerve cord development (Fig. 4A, Table 1).

**Table 1:** Quantification of *Aae fra* knockdown phenotype penetrance and severity.

	AnteriorWT	Mild	Strong	PosteriorWT	Mild	Strong
**Control**	91 (100%)	0 (0%)	0 (0%)	91(100%)	0 (0%)	0 (0%)
***fra*** ** siRNA-A**	22 (29%)	26 (35%)	27 (36%)	15 (20%)	22 (29%)	38 (51%)

Embryos stained with anti-acetylated tubulin were scored at 54 hrs. post-injection of *fra* siRNA-A or scrambled control siRNA. The number and percentage of total segments bearing wild-type (WT), mild, or strong phenotypes in the anterior and posterior commissures are reported. Mild phenotypes correspond to thinning commissures, and severe phenotypes correspond to near or complete absence of commissural axons.

It should be noted that the penetrance and severity of the *Aae fra* knockdown phenotype are higher than that reported for the *Drosophila fra* null, in which only 12% of the anterior commissures and 43% of the posterior commissures are reportedly thin or absent [27]. In fact, in embryos in which CNS transcripts are nearly depleted, the *Aae fra* knockdown phenotype (Fig. 4B,C) bears strong resemblance to the *Drosophila commissureless* phenotype, in which commissure formation is entirely blocked [40]. These results suggest that Net-Fra signaling may play a more critical role in formation of the *Ae. aegypti* ventral nerve cord, and that the guidance cues postulated to compensate for loss of Net-Fra signaling in *Dr. melanogaster* [29] may not be present in mosquitoes. These observations suggest that further analysis of embryonic nerve cord development in mosquitoes may uncover underlying differences between *Dr. melanogaster* and mosquito nervous system development. In support of this concept, our ongoing analysis of *semaphorin* knockdown in *Ae. aegypti* suggests that the function of this gene in nerve cord development has evolved in insects (data not shown).

### Developmental Genetics in Vector Mosquitoes

Although we have made great advances in understanding developmental genetics in *Drosophila,* comparatively little is known about the genetic basis for development in mosquitoes and other arthropods. In this investigation, we examined the role of Fra during development of two vector mosquitoes. Expression of *fra* in the developing ventral nerve cord was found to be conserved between the two mosquitoes and *Dr. melanogaster.* However, the results of this investigation, the first targeted knockdown of a gene during vector mosquito embryogenesis, illustrate that although Fra plays a critical role during development of the *Ae. aegypti* ventral nerve cord, mechanisms regulating embryonic commissural axon guidance may have evolved in distantly related insects. This is a somewhat unexpected finding given the many similarities in insect CNS development that have been observed (for example, see [34], [41]). Given these findings in *Ae. aegypti,* it would also be interesting to apply the siRNA-mediated knockdown strategies utilized here to *An. gambiae* and to formally assess the function of *Aga fra.*

Characterizing the function of additional developmental genes in mosquitoes is critical. To date, expression patterns of only a handful of mosquito developmental genes [9], [10], [11], [12], [13], [14], [15], [16] have been reported. Adelman et al. [13] showed that control sequences for one of these genes, *nanos* [11], demonstrated promise as part of a transposable element-based gene drive system that may be used to spread and fix antipathogen effector genes in natural populations. Their investigations illustrate the exciting potential for the application of evo-devo approaches in efforts to develop strategies for vector control. The methodologies used in this investigation, in particular the siRNA-mediated knockdown strategy for functional analysis of developmental genes in *Ae. aegypti* embryos, will broaden and enhance these efforts.

## Materials and Methods

### Ethics statement

This study was performed in accordance with the recommendations in the Guide for the Care and Use of Laboratory Animals of the National Institutes of Health. The animal use protocol was approved by the University of Notre Dame Institutional Animal Care and Use Committee (Study # 11-036).

### Mosquito Rearing, Egg Collection, and Fixation

The *Ae. aegypti* Liverpool-IB12 (LVP-1B12) strain and *An. gambiae* (M Form) were used in these investigations. Procedures for mosquito rearing and egg collection [22], [42], which was performed at 26°C, have been described. *Ae. aegypti* embryos were fixed as described [20]. *An. gambiae* embryos were fixed using a comparable procedure, except that eggs were fixed at room temperature.

### Immunohistochemistry

Immunohistochemistry was performed as described [19]. Anti-acetylated tubulin (Zymed, San Francisco, CA) was used at a concentration of 1∶100, and HRP-conjugated secondary antibodies (Jackson Immunoresearch, West Grove, PA) were used at a final concentration of 1∶200.

### *In situ* hybridization

Riboprobes corresponding to *Aae fra* (AAEL014592) and *Aga fra* (AGAP006083) were synthesized according to the Patel [43] protocol. *In situ* hybridization was performed as previously described [23].

### RNA interference

Knockdown was performed through embryonic microinjection of siRNAs targeting *Aae fra.* siRNA design and microinjection were performed as described [21]. The following siRNAs were synthesized by Dharmacon RNAi Technologies (Lafayette, CO): siRNA-A sense: CCA GAT GGG TAT GGG AGA T and antisense: GGT CTA CCC ATA CCC TCT A (corresponding to base pairs 2011–2032 of *Aae fra*) and siRNA-B sense: TCC ATA CAC CTA CGA AGG A and antisense: AGG TAT GTG GAT GCT TCCT (corresponds to base pairs 3862–3883 of *Aae fra*). A scrambled version of siRNA-A was used as a control: sense GAT TAG ACG AAT ACC ACT A and antisense: CTA ATC TGC TTA TGG TGA T. siRNAs were injected at a concentration of 6 ug/uL.

Measurement of knockdown effectiveness was determined through *in situ* hybridization (see above) and through qRT-PCR. qRT-PCR was performed as previously described [44]. In short, total RNA was extracted from ∼30 pooled siRNA-microinjected mosquito embryos using Trizol (Invitrogen, Carlsbad, CA). cDNA was prepared with the High Capacity RNA to cDNA Kit (Applied Biosystems, Foster City, CA), which includes a blend of random and oligo(dT) primers, according to the manufacturer's instructions. Real-time quantification was performed using the SYBR Green I PCR kit (Applied Biosystems, Foster City, CA) in conjunction with an Applied BioSystems Step One Plus Real-Time PCR System. Primer sets for *Aae fra* were: For 5′ GCG ACC CAA CAC TCA ATA TG 3′ and Rev 5′ GTC GTA GGA TAC CGT GAG AT 3′. Primer sets for the housekeeping gene *rpS17*, which was included as the internal standard for data normalization as previously described [44], were as follows: For 5′ AGA CAA CTA CGT GCC GGA AG 3′ and Rev 5′ TTG GTG ACC TGG ACA ACG ATG 3′. Three independent biological replicates were conducted, and all PCR reactions were performed in triplicate. Quantification of results was accomplished by standardizing reactions to *rpS17* levels, and then using the ΔΔCt method as described [45]. Results were expressed as fold-difference compared with the scrambled control-injected embryos. qRT-PCR data from replicate experiments were statistically analyzed with the Student T Test.
